# Calponin is expressed by subpopulations of connective tissue cells but not olfactory ensheathing cells in the neonatal olfactory mucosa

**DOI:** 10.1186/1471-2202-8-74

**Published:** 2007-09-18

**Authors:** Mercedes Tomé, Edina Siladžic, Alessandra Santos-Silva, Susan C Barnett

**Affiliations:** 1 Division of Clinical Neuroscience, Beatson Institute, University of Glasgow, Garscube Estate, Switchback Road, Glasgow, G61 1BD, UK

## Abstract

**Background:**

Debate has been ongoing on the relative merits of olfactory ensheathing cells (OECs) and Schwann cells as candidates for transplant-mediate repair of CNS lesions. Both glial cells exhibit similar molecular and cellular properties and to date there has been no antigenic marker identified that can clearly distinguish the two cell types. This inability to distinguish between the two cells types prevents confirmation of a controversial statement that cultures of OECs are contaminated with Schwann cells. Recently, proteomic analysis of foetal OECs and adult Schwann cells identified an actin-binding protein, calponin, as a specific marker for OECs. However, at the same time a recent report suggested that adult OECs do not express calponin. It was not clear if this discrepancy was due to methodology, as cells had to be treated with proteinase K to maximize calponin staining or developmental differences with only foetal/neonatal OECs expressing calponin. For this reason we have examined calponin expression in the peripheral olfactory system of embryonic and neonatal rats *in vivo *and from cells *in vitro *to assess if calponin is expressed in a developmental manner.

**Results:**

In this study we show that: i) proteinase K pretreatment had no effect on calponin staining in both OECs and Schwann cells. ii) calponin immunoreactivity was not expressed by embryonic or neonatal OECs *in vitro *and *in vivo *although connective tissue from the olfactory mucosa was strongly positive in neonatal rats but not embryonic rats, iii) calponin expression in the olfactory mucosa was heterogeneous, defining subpopulations of connective tissue cells iv) using functional confrontation assays between OECs or Schwann cells with astrocytes, calponin was expressed heterogeneously by astrocytes.

**Conclusion:**

It is concluded that calponin is heterogeneously expressed by neonatal mucosal connective tissue but not expressed by neonatal OECs, embryonic OECs, and neonatal Schwann cells. Furthermore, we propose that calponin is not a specific marker for OECs generated from any developmental age.

## Background

Olfactory ensheathing cells (OECs) are the glial cells of the primary olfactory system [1]. The olfactory system is comprised of both PNS and CNS tissue and known for its ability to support neurogenesis throughout life [2,3]. It is believed that the role of OECs in the olfactory system is to direct the newly generated axons to their correct position in the CNS environment of the olfactory bulb [2]. OECs have become cells of interest due to their potential as candidates for transplant-mediated repair of CNS pathologies, such as spinal cord injury and multiple sclerosis [4-7]. Several studies have demonstrated that transplanted OECs can in the right environment, myelinate experimentally created demyelinated axons [8-10]. However, it has been suggested that myelination seen after transplantation of OECs is due to contaminating Schwann cells [11] or by host Schwann cells invading the lesion site [12-14].

It has been extremely difficult to confirm if OECs can indeed myelinate axons *in vivo *due to the inability to distinguish OECs from Schwann cells using cell markers. This is because they share many characteristics in common including antigenic and morphological phenotype [15]. Even more striking is the similarity in their myelinating phenotype after transplantation into a CNS lesion where the myelin formed is typical of PNS type myelin [8,9]. Furthermore the transcriptional regulation of these cells during myelination is very similar [16]. However, although these cells share many antigenic characteristics, they do not have identical biological properties. For example, this is seen in the different way they interact with astrocytes either *in vitro *[17] using "confrontation assays" or after transplantation *in vivo *[17-19]. In these confrontation assays Schwann cells form a boundary with astrocytes and induce characteristics of the stress response. These include an increase in astrocytic size and GFAP expression levels. OECs do not form this cellular boundary with astrocytes nor induce this stress response in astrocytes [17,20]. Recent data suggest that Schwann cells secrete a different heparin sulphate proteoglycan profile to OECs and it is this factor(s) that mediates this astrocytic response [21]. These studies indicate that OECs may be more favorable than Schwann cells in transplant-mediated repair strategies. However, it is the lack of a specific OEC or Schwann cell marker that has lead to problems in identifying the cells after transplantation and has caused discrepancy in deciding which glial cell is optimal for promoting CNS repair [5,22,23].

Recently, proteomic analysis of foetal OECs and adult Schwann cells has identified calponin, an actin-binding protein, as a specific marker for OECs [24]. This has led to some conflicting reports regarding the expression of calponin in the adult peripheral olfactory system [11,25]. In the study from Boyd et al., [24] it was suggested that OECs were the only glial cell to express calponin. In a later study by Ibanez et al., [25] it was demonstrated a lack of calponin expression in cultured OECs and *in vivo *within the adult rat peripheral system. However, it was not clear if this discrepancy was due to methodology in calponin staining as it has been reported that cells had to be treated with proteinase K to maximize staining [11]. Discrepancy could also be due to the different developmental age of rats used in both studies. In our study we show that in the presence and absence of proteinase K pretreatment punctate, nuclear calponin immunoreactivity could be detected in both neonatal OECs and neonatal Schwann cells suggesting non-specific attachment of the secondary antibody in both cell types. Interestingly, using confrontation assays, which have been the only way to identify differences between OECs and Schwann cells *in vitro*, we observed intense, fibrillar calponin immunoreactivity associated only with astrocytes. This intense staining was similar to that seen for fibroblasts. Calponin immunoreactivity was also detected in astrocytes generated from P1 neurosphere cultures. As demonstrated for *in vivo *immunohistochemistry of the rat adult peripheral olfactory system, calponin immunoreactivity was not observed in neonatal OECs but heterogeneously colocalised with markers of connective tissue in the lamina propria (LP) defining discrete areas. Furthermore embryonic tissue was generally devoid of any calponin staining suggesting developmental changes in expression of calponin in connective tissue in the olfactory system. These data suggest that the actin-binding protein calponin may be a marker for subpopulations of olfactory mucosa connective tissue cells but is not a specific marker for embryonic or neonatal OECs.

## Results

### Neonatal OECs do not express calponin in the olfactory mucosa *in vivo*

**Figure 1:**
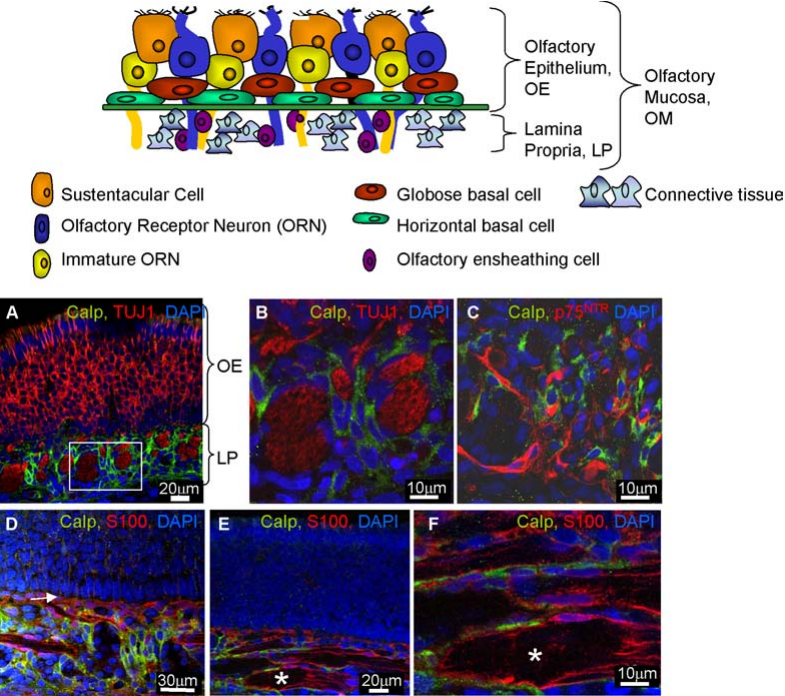
**OECs in the neonatal olfactory system *in vivo *do not express calponin**. The schematic in Figure 1 illustrates the histology of the olfactory mucosa (OM). The OM can be divided into two parts, the olfactory epithelium (OE) and the lamina propria (LP). The OE contains mature and immature olfactory receptor neurons (ORNs) and their support cells (sustentacular cells) and two types of potential stem cells; globose basal cells and horizontal basal cells. The LP contains extensions of ORN axons which are ensheathed by OECs. Supporting the glia and axons in the LP is loose connective tissue which contains fibroblasts, Bowman glands and blood vessels. **A-B) **Calponin did not colocalise with an ORN marker (TUJ1 positive-red, calponin-green and DAPI-blue). Figure 1B is a higher magnification of the boxed area in Figure 1A. Calponin did not colocalise with OECs as defined by expression of **C) **p75^NTR ^(p75^NTR ^-red, calponin-green, DAPI-blue) or **D, E, F) **S100 (S100-red, calponin-green, DAPI-blue, * nerves). Figure 1F is a higher magnification of Figure 1E. All images were obtained using confocal microscopy.

The schematic in Figure 1 illustrates the histology of the olfactory mucosa (OM) and the cellular composition of the olfactory epithelium (OE) and lamina propria (LP). Olfactory receptor neurons (ORNs) extend their axons from the OE to the LP where they coexist with the OECs and the supportive connective tissue. Within this tissue may also be Schwann cells ensheathing nerves innervating blood vessels. In Figure 1A it can be seen that the calponin immunoreactivity was only detected in the LP of neonatal OM. Olfactory receptor nerves were intensively inmunoreactive for the neuronal marker TUJ1, and were surrounded by cells immunoreactive for calponin (Figure 1A, B). No colabelling was detected between calponin and TUJ1, with calponin positive cells and their nuclei clearly distinguishable from the nerves (Figure 1B). Since OECs are known to typically surround ORN axons, double immunofluorescence for calponin and the OEC markers S100 and p75^NTR ^was performed in the neonatal tissue (Figure 1C–F). As expected, S100 and p75^NTR ^positive OECs were located just underneath the basal layer of the OE (Figure 1D, arrow) and surrounding the nerves (Figure 1E, F, asterisk). Calponin immunoreactivity was located close to the S100 and p75^NTR ^positive OECs but no colocalisation was observed with either OEC markers (Figure1C–F). Even though Schwann cells (identified by the same markers as OECs) may be present in the olfactory mucosa, we did not observe any cells immunoreactive for both p75^NTR ^and calponin indicating lack of calponin positive OECs or Schwann cells. This staining was specific as control reactions lacking the primary antibody did not result in any immunoreactivity. The absence or presence of proteinase K also has no effect on the pattern of immunoreactivity.

### Calponin is expressed by cells of the neonatal connective tissue in the lamina propria *in vivo*

**Figure 2:**
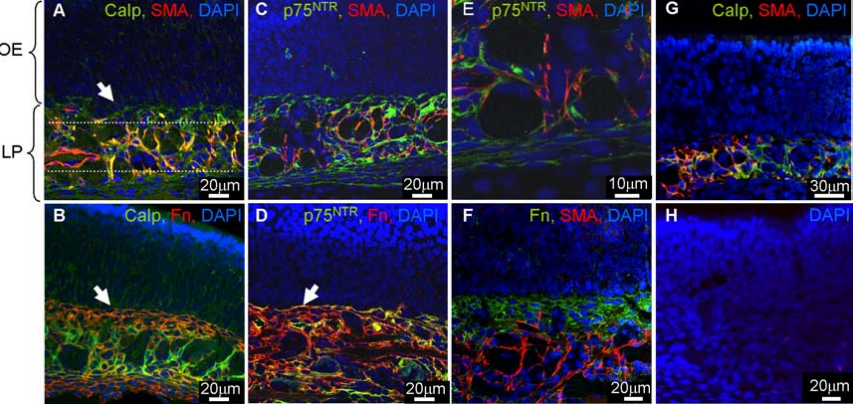
**Connective tissue in the lamina propria of theolfactory system *in vivo *expresses calponin**. Sections of olfactory mucosa were immunolabeled with antibodies to connective tissue and glia together with calponin and visualized using confocal microscopy. **A, B**) Calponin immunoreactivity was associated with markers of connective tissue fibroblast-like cells, Fn (red) and α-SMA (red). Dashed area represents the central part of the LP associated with α-SMA immunoreactivity and the arrow indicates the area adjacent to the basal part of the OE. **C, D**) To identify connective tissue, sections were immunolabeled with α-SMA and Fn together with p75^NTR^. OECs (p75^NTR^-green) did not express α-SMA. Figure 2E is a higher magnification Figure 2C. **F**) Double immunofluorescence with both connective tissue markers Fn and α-SMA. The α-SMA positive cells, located mainly in the central part of the LP, do no colocalise with Fn, that is intensively expressed in the boundary between LP and OE. **G**) Similar staining patterns were obtained when proteinase K was excluded from the protocol. **H**) No tissue staining occurred in control sections when the primary antibody was excluded (DAPI, blue).

To identify the cells that express calponin in the LP from neonatal rats, we carried out double immunofluorescence with antibodies to calponin together with markers of connective tissue namely fibronectin (Fn) and alpha smooth muscle actin (α-SMA). Connective tissue cells immunoreactive for α-SMA were mainly located in the central region of the LP and associated with the olfactory nerves (Figure 2A as indicated by dashed lines). Double staining for α-SMA and calponin showed colabelling of both markers in this same region (Figure 2A). α-SMA positive staining that lacked calponin in this region was associated with blood vessels. To remove the possibility that the calponin positive cells were OECs immunolabeled with α-SMA as has been suggested by Jahed et al., [26] double immunofluorescence for α-SMA and both S100 and p75^NTR ^was performed. No colocalisation was observed between α-SMA and both OEC markers further supporting our evidence for a lack of expression of calponin and α-SMA in neonatal OECs (Figure 2C, E, data not shown for S100). However, not all the cells immunoreactive for calponin colabelled with α-SMA suggesting there was another cell population that expressed calponin in the LP. Calponin positive cells that lacked immunoreactivity to α-SMA were seen in areas closer to the basal part of the OE (Figure 2A, arrow). Double immunofluorescence for Fn and calponin marked in general a different area of immunoreactivity than α-SMA in the LP (compare Figure 2A and 2B at arrow). Fn colocalised with some calponin in the area directly underneath the basal layer of the olfactory epithelium (Figure 2B, arrow). This suggests that another connective tissue cell co-expresses Fn and calponin but lacks α-SMA (Figure 2F). A proportion of the Fn positive cells which lacked calponin must be OECs as they coexpress p75^NTR ^(Figure 2D, arrow). Similar staining patterns were obtained when proteinase K was either included or excluded from the protocol (Figure 2G). Once more, control experiments in which the primary antibody was omitted, were negative (Figure 2H).

### Calponin is not expressed in the embryonic olfactory mucosa *in vivo*

**Figure 3:**
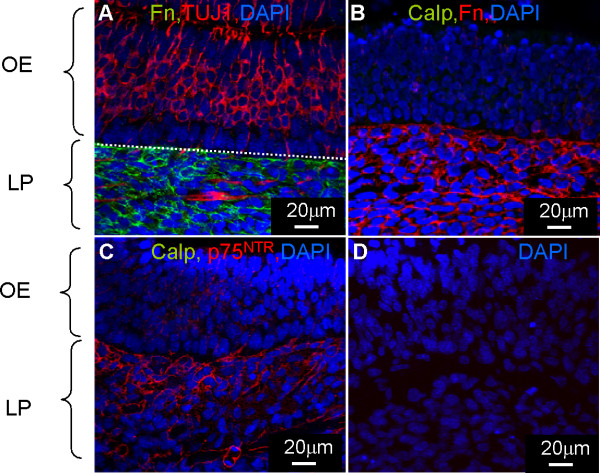
**Calponin is not expressed in embryonic tissue**. The olfactory mucosa was immunolabeled with antibodies to identify **A**) ORNs (TUJ1-red, Fn-green, DAPI-blue), **B**) connective tissue (Fn-red, calponin-green, DAPI-blue), **C**) OECs (p75^NTR^-red, calponin-green, DAPI-blue) and **D**) control tissue omitting the primary antibody. Images were obtained using the confocal microscope. The embryonic olfactory mucosa lacked any calponin immunoreactivity even though it expressed other markers which defined the OECs and connective tissue seen in the P7 olfactory mucosa.

Embryonic olfactory mucosa was immunostained in the presence and absence of proteinase K with the same markers used in neonatal OM tissue to reveal possible age-related calponin expression in OECs. Embryonic olfactory neurons and their axons were imunoreactive for TUJ1 (Figure 3A). p75^NTR ^positive OECs and Fn positive connective tissue were detected in the LP as seen for neonatal tissue (Figure 3A–C). No immunoreactivity for calponin could be detected in any region of the embryonic olfactory mucosa. Furthermore inclusion of proteinase K did not reveal calponin staining. The lack of calponin in the embryonic tissue suggests that this protein is expressed later on in the development in the olfactory connective tissue. Control experiments in which the primary antibody was omitted were negative (Figure 3D).

### Calponin is not expressed by purified OECs and Schwann cells *in vitro*

**Figure 4:**
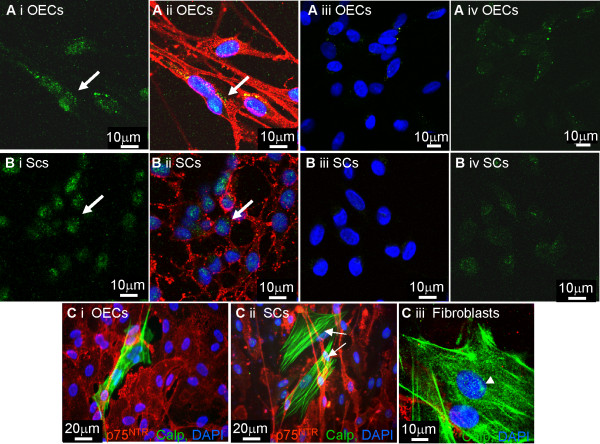
**Purified OECs and Schwann cells *in vitro *do not express calponin**. OECs and Schwann cells were immunolabeled with calponin in the presence and absence of proteinase K and no differences were found. **A, B**) OECs and Schwann cells were immuolabelled with p75^NTR ^(red) and calponin (green). Greater than 99% of the cells immunolabeled with p75^NTR ^(red) but a background level of green, punctate immunoreactivity could be seen associated with the nuclei (Ai, ii, Bi, ii, arrows). When primary antibody was omitted weaker punctate staining could just be detected (Aiii, iv, Biii, iv) **C**i, ii) OEC and Schwann cell cultures containing rare fibroblast contaminants (arrows) were immunolabeled with calponin (green) and p75^NTR ^(red). It can be seen that the calponin immunoreactivity in the fibroblasts was strongly fibrillar and distinct from the non-specific attachment of the secondary antibody detected in OECs and Schwann cells. **Ciii) **illustrates a higher power image of contaminating fibroblasts which although expressed fibrillar calponin immunoreactivity, also expressed the punctate nuclear background immunoreactivity seen in OECs and Schwann cells. Images for Figures A, B and Ciii were obtained using a confocal microscope and images for Figures Ci, ii were obtained using a fluorescent microscope.

In Figure 4A, B, purified cultured OECs and Schwann cells were plated onto coverslips and labeled with calponin together with p75^NTR^. Since it has been reported that calponin immunoreactivity in glia was maximized by pretreatment with proteinase K [11], we compared methods in which pretreatment was omitted. In the presence and absence of proteinase K pretreatment both OECs and Schwann cells expressed weak, nuclear, background levels of calponin immunoreactivity (Figure 4Ai, ii, Bi, ii, illustrates cells treated with proteinase K but similar results occurred for cells not treated). However, when the primary antibody was omitted this staining was reduced but could still be detected suggesting that this nuclear background staining is non-specific attachment of the secondary antibody (Figure 4Aiii, iv and 4Biii, iv). Also, note the similar nuclear background staining (arrowhead) in the fibroblasts which express strong fibrillar calponin immunoreactivity (Figure 4Ci–iii, arrow). These fibroblast-like cells were devoid of p75^NTR ^immunoreactivity.

### Calponin is expressed by fibroblast-like cells purified from olfactory tissue and sciatic nerve

**Figure 5:**
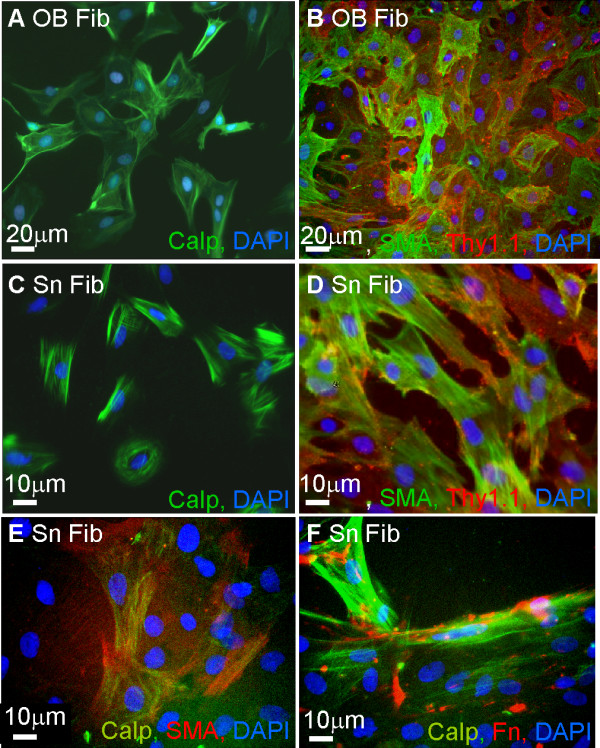
**Fibroblast- like cells from the olfactory system and sciatic nerve express calponin**. Neonatal olfactory bulb (A, B) and the sciatic nerve (C, D) were dissociated to obtain purified fibroblast-like cell cultures respectively. **A, C) **Purified fibroblast-like cultures from the olfactory bulb (A) and sciatic nerve (C) expressed calponin immunoreactivity (green) with typical fibrous appearance. **B, D**) Purified fibroblast-like cells immunolabeled for the connective tissue markers Thy1.1 (red) and α-SMA (green) in both olfactory bulb (B) and sciatic nerve (D). Colocalisation of both markers with calponin was shown for sciatic nerve fibroblasts. All images were obtained using a fluorescent microscope.

To confirm the connective tissue origins of the contaminating calponin positive fibroblast-like cells observed in the OEC and Schwann cell cultures, cultures enriched for non glial cells were generated from neonatal olfactory bulb and sciatic nerve by incubating the cultures in DMEM containing 10% FBS (Figure 5A–B). Cultured cells exhibited a fibroblast-like morphology and expressed strong immunoreactivity for calponin with a characteristic fibrillar appearance. These fibroblast-like cultures were heterogeneous for the expression of α-SMA and Thy1.1 that resembles the heterogeneity we observed in the LP of the *in vivo *olfactory mucosa (Figure 5B, D). Double immunofluorescence revealed colocalisation of calponin with α-SMA and surface fibronectin, supporting the non-glial origin of the calponin immunoreactivity observed in the purified OEC and Schwann cell cultures (Figure 5E, F).

### Calponin expression in confrontation assays is associated with astrocytes

**Figure 6:**
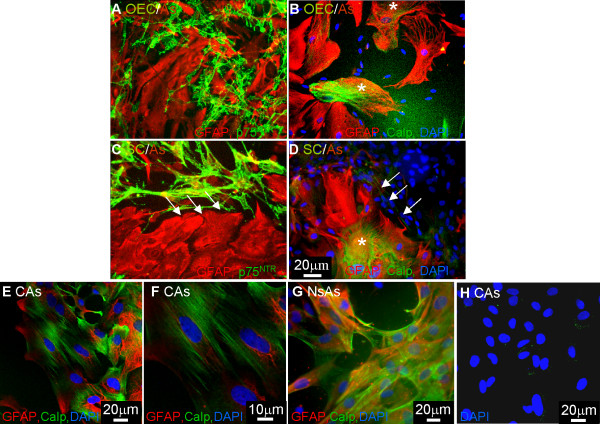
**Confrontation assays illustrate astrocytes express calponin. **Confrontation assays were set up between OECs/astrocytes (A, B) and Schwann cells/astrocytes (C, D). **A**) OECs mingled with astrocytes while **B**) Schwann cells formed a boundary with astrocytes. Astrocytes expressed GFAP (red) and OECs or Schwann cells expressed p75^NTR ^(green). **B, D**) When these cultures were labeled with calponin (green), GFAP (red) and DAPI for nuclei of the Schwann cells or OECs (blue) it could be seen that both OECs and Schwann cells lacked calponin expression (green immunoreactivity alone) but astrocytes expressed calponin (red and green immunoreactivity). **E-G**) Astrocytes generated from P1 cortex and P1 neurospheres were immunolabeled with antibodies to GFAP (red) and to calponin (green). Neonatal cortical astrocytes (CAs) were over 98% pure as assessed by GFAP expression (E) and expressed fibrillar calponin heterogeneously (E-F). Astrocytes generated from neurospheres (NsAs) by differentiation in DMEM- 10% FBS also expressed calponin heterogeneously in culture (**G**). **H**) No cell staining occurred in control cultures when the primary antibody was excluded (DAPI, blue). Images for Figures 6A-D, G were collected using the fluorescent microscope and images for Figures 6E, F, H were collected using confocal microscopy.

It has been suggested that cultures of OECs are contaminated with Schwann cells [11]. A major functional difference between OECs and Schwann cells can be seen in their ability to interact with astrocytes, with OECs clearly mingling with astrocytes and Schwann cells and astrocytes forming boundaries [18,20,21]. These differences are believed to be due to a differential FGF/heparin sulphate proteoglycan signaling cascade [21]. To illustrate the purity of our OEC preparation, we set up these confrontation assays and immunolabeled the cells with GFAP, p75^NTR ^and calponin. These assays take 10–14 days for the cells to meet and are carried out in DMEM -10% FBS. As Schwann cells take up Brdu in DMEM-10% FBS (data not shown) we believe this would be enough time for any possible contaminating Schwann cells to increase in number. Therefore, we would expect any contaminating Schwann cells in these confrontation assays to affect boundary formation. Due to the similarity in the class specific secondary antibody for calponin and p75^NTR^, OECs and Schwann cells in these cultures were detected by the blue DAPI nuclei associated with unlabeled cells, and sister coverslips were immunolabeled with p75^NTR ^and GFAP. In Figure 6A, C confrontation assays between Schwann cells and OECs with astrocytes can be seen. OECs mingled with astrocytes (Figure 6A) while the Schwann cells formed a boundary (Figure 6C, arrows). To our surprise, calponin staining could be seen to be associated with astrocytes and not OECs or Schwann cells (Figure 6B, D, asterisk). This difference in the way Schwann cells interact with astrocytes in confrontation assays when compared to OECs gives support to the view that there is no Schwann cell contamination in OEC cultures after at least 2 weeks.

To confirm cultured astrocytes express calponin, we generated two types of astrocytes, cortical astrocytes (CAs) and astrocytes differentiated from neurospheres (NsAs). Cortical astrocytes were over 98% pure as assessed by GFAP expression and these astrocytes express calponin heterogeneously (Figure 6E–F). The calponin was fibrillar and similar to that seen for fibroblasts. Astrocytes generated from P1 neurospheres by incubation in DMEM containing 10% FBS also expressed fibrillar calponin in a heterogeneous manner (Figure 6G). Control cultures without primary antibody were negative although a weak background staining was also detected as seen for OECs and Schwann cells (Figure 6H).

## Discussion

OECs and Schwann cells have been proposed as candidates for transplant mediated repair strategies of spinal cord injury. Previous studies have shown that they share a number of remarkably similar phenotypes, and growth factor responses [15,22,23,27]. It is only recently that molecular studies have identified differences between these two cell types specifically in their interaction with astrocytes [17-19,21,28]. Furthermore, evidence suggests that other cell types, eg. fibroblast-like cells, previously rejected as contaminating cells, may have a role in enhancing repair. Indeed, studies are coming to light that suggest that the extracellular matrix produced by these contaminating fibroblast-like cells may also have an important role in mediating repair [29-31]. For this reason it is quite important to identify the individual cells used in transplant-mediate repair to assess their contribution to resulting regeneration. To date there has been no markers identified that specifically labels OECs or distinguishes them from Schwann cells. Recently it has been proposed that calponin is a specific marker which is able to distinguish OECs from Schwann cells [11,24]. Using this marker to immunolabel cultures of OECs, a mixture of p75^NTR ^positive/calponin positive and p75^NTR ^positive/calponin negative cells were seen and it was proposed that cells lacking calponin immunoreactivity was attributed to contaminating Schwann cells. For this reason the authors suggest that any myelination seen *in vivo *after OEC transplantation was solely due to contaminating Schwann cells [11]. This hypothesis has been challenged with evidence demonstrating a lack of calponin staining in the adult rat olfactory system [25]. In this report only adult tissue was examined as it was suggested autologous transplantation is likely to be the basis of clinical translation studies, but an ongoing clinical trial in China is using embryonic olfactory tissue as their source of tissue [32]. Furthermore, it is necessary to examine calponin expression in neonatal OECs and embryonic tissue to allow comparison with the many cell transplantation studies in the literature that has used neonatal OECs and with the original proteomic analysis which used foetal OECs [24]. A study on neonatal and embryonic tissue allows comparison to be made of calponin expression during development and any possible age-related expression in OECs.

In our study we demonstrate that calponin immunoreactivity may be detected in both OECs and Schwann cells in culture although the expression is not typical of calponin immunoreactivity reported in the literature. The calponin immunoreactivity for the glial cells was punctate and nuclear and not fibrillar as seen in muscle cells [33] or in fibroblast as demonstrated in this study (Figure 5) and by others [25]. However, omitting the primary antibody reduced but did not remove this punctate staining around the nucleus. On the other hand, we found similar nuclear, punctate immunoreactivity in strongly positive fibroblasts which suggests the punctate staining seen in Schwann cells and OECs is more likely to be background non-specific staining. We initially hypothesized that the nuclear staining detected in the OECs and Schwann cells may be due to proteinase K treatments (recommended for tissue immunocytochemistry by the antibody supplier) but we had identical staining in the presence and absence of proteinase K with methanol fixation. In support of this, all of our immunohistochemistry of tissue sections was carried out with proteinase K pretreatment, but still did not reveal calponin staining in OECs. Thus, our data using neonatal and embryonic tissue support the results from adult olfactory tissue that calponin is not a marker for OECs [25].

Our *in vivo *studies identified strong calponin immunoreactivity in the connective tissue of the LP of neonatal rats. Heterogeneous antigenic expression was detected in tissue sections and purified cultured cells of the classical connective tissue markers, eg. antibodies to fibronectin, α-SMA, [34-36] and Thy1.1 [37] (for fibroblasts and muscle cells), together with the calponin antibody. Section of neonatal mucosa showed regional differences in connective tissue markers suggesting that subpopulations of fibroblasts exist in the LP. The middle section of the LP contained α-SMA immunoreactivity while the area adjacent to the OE expressed mainly fibronectin and Thy1.1. A proportion of this α-SMA immunoreactivity could be attributed to smooth muscle cells lining blood vessels but the distribution of α-SMA immunoreactivity was too large to be due to these cells alone. In cell cultures prepared from the olfactory bulb and sciatic nerve these markers identified heterogeneously labeled fibroblast-like cells. This is consistent with reports that α-SMA, calponin and Thy1.1 characterize subpopulations of fibroblasts [34,36,37]. Thus, our data suggest that connective tissue from the olfactory mucosa is comprised of several subclasses of fibroblasts. Although fibronectin labels connective tissue we have seen coexpression with p75^NTR ^confirming reports that OECs express fibronectin [38] and that fibronectin expressed in the olfactory nerve layer of the olfactory bulb is likely to be due to OECs [39]. However, it has been suggested that neural crest precursor cells can generate Schwann cells and endoneurial fibroblasts raising the possibility that this type of cell may exist in the developing olfactory mucosa which transiently coexpress p75^NTR ^and fibroblast markers [40]. Interestingly, the expression of calponin in the connective tissue of neonatal LP may reflect developmental changes, since the olfactory system of embryonic rats lacked calponin immunoreactivity. This finding may have relevance for CNS repair as mixed olfactory tissue is being proposed as a more potent reparative cell mix for transplant-mediated repair of spinal cord injury [29-31]. To be able to identify and work with these different cells types may lead to a better understanding of their role in CNS repair.

It has also been suggested that preparations of cultured OECs are contaminated with Schwann cells [11]. OECs used in this study are prepared from neonatal olfactory bulbs using the O4 antibody and fluorescence activated cell sorting [15] and are over 98% p75^NTR ^pure. Although we have seen lack of calponin staining in either of our Schwann cells or OECs preparations, we believe that our OEC preparations are devoid of Schwann cells due to their inability to form any boundary in confrontation assays [17,20]. To support this data we carried out confrontation assays with OECs/astrocytes and Schwann cells/astrocytes and immunolabeled the cells with calponin and GFAP. Once more we saw the differential migratory behavior of OECs and Schwann cells when in contact with astrocytes and unexpectedly calponin immunoreactivity was associated with astrocytes. Immunolabeling pure cultures of astrocytes from two different sources supported the finding that astrocytes express calponin. Other reports have demonstrated calponin expression in the brain associated with hippocampal and cerebellar neurons as well as radial glia, Bergman glia, glia limitans and mature astrocytes [41,42]. However these studies used an antibody to the acidic isoform of calponin. Calponin is developmentally expressed as 3 isoforms; α,β and γ. The basic α and β isoforms have molecular weights of 34 kD and 29 kD respectively. An acidic isoform of calponin has also been reported with a molecular weight of 36 kD [43]. It is this acidic isoform that has been reported to be expressed in a range of tissue and not just muscle cells [35,42]. The antibody we used in this study is the same antibody described by Rizek and Kawaja [11] and the same clone (different supplier) as described by Ibanez et al., [25] which recognizes the basic calponin isoform. This suggests the basic calponin isoform can be expressed by astrocytes *in vitro*. Calponin expression in these astrocytes was heterogeneous and may reflect subsets of astrocytes or a differential expression during the cell cycle [44,45].

## Conclusion

Our data suggest that: i) calponin expression in the olfactory mucosa was heterogeneous defining subpopulations of connective tissue cells; ii) calponin did not label OECs in embryonic and neonatal tissue; iii) neonatal OECs and Schwann cells express weak, punctate, nuclear calponin immunoreactivity *in vitro*, which correlates with a non-specific staining also seen in astrocytes and fibroblasts; iv) strong fibrillar basic calponin was expressed heterogeneously by astrocytes *in vitro*. These data demonstrate that calponin is not a specific marker for rat OECs generated from any developmental age.

## Methods

### Cell culture

Primary neural cells were purified and cultured as previously described. Neonatal OECs were purified from the olfactory bulb of 7-day-old Sprague-Dawley rat pups using the O4 antibody, anti-galactocerebroside and the fluorescent activated cell sorter (FACSVantage Becton Dickenson, [14]. OECs were cultured in defined medium DMEM (DMEM-BS, [46]) with 5% FBS on poly-L-lysine (PLL, 13 μg/ml; Sigma) coated 25 cm^2 ^flasks, and further supplemented with fibroblast growth factor 2 (FGF2, 500 ng/ml; Peprotech, London, UK), heregulin (hrgβ1, 50 ng/ml; R&D Systems Europe Ltd., Abingdon, UK), forskolin (10^-6^M; Sigma) and astrocyte conditioned medium (ACM, [47]) which has been shown to be a potent mitogen for promoting prolonged growth of p75 neurotrophin receptor (p75^NTR^) expressing OECs [48].

Schwann cells were obtained from sciatic nerves of 7-day-old Sprague-Dawley rat pups and purified using a modification of the method described by Brockes and colleagues [49]. The modification involved an initial flick of the flask after one day in culture to preferentially select for Schwann cells, followed by incubation with Thy1.1 antibody (1:50 supernatant; Sigma, Dorst, UK) and rabbit complement (1:6; Harlan Sera-lab Ltd, Loughborough, UK) to reduce contamination by fibroblasts. Cells were plated on PLL (13 μg/ml, Sigma) coated flasks and maintained in DMEM-10% FBS supplemented with forskolin (10^-6 ^M; Sigma) and hrgβ1, (20 ng/ml; R&D Systems Europe Ltd, Oxon, UK).

Astrocytes were purified from the cortex as previously described [18,47]. The cells were maintained in DMEM containing 10% FBS on PLL coated flasks. ACM was collected from confluent flasks of astrocytes by incubating the cells in 10 mls of serum free DMEM [47] as described in Lakatos et al., [18] for 48 hrs before collecting, spinning and filtering [47]. Neurospheres were generated as described by Reynolds and Weiss [50] from P1 striatum, plated in neurobasal medium containing B27 supplements (Invitrogen, Paisley, UK). Neurosphere astrocytes were established by plating neurospheres onto PLL coated coverslips and incubating them in DMEM containing 10% FBS for a week.

### Immunocytochemistry

Cell purity was assessed by labeling the cells with GFAP (glial fibrillary acid protein) antibody for astrocytes, and p75^NTR ^antibody for OECs and Schwann cells [51]. All cultures were greater than 98% pure. The cells were maintained in culture at 37°C in 7% CO_2 _for no longer than 1 month and were refed twice a week. For immunofluorescence the cells were passaged onto PLL coated 13 mm coverslips. To study cell surface antigen, cells were incubated with the primary antibody for 30–40 min at room temperature (RT) followed by the class specific antibody for 30 min at RT. Intracellular antibodies were incubated on cells permeabilised using 100% ice cold methanol and placed in the freezer for 15 min followed by their class specific antibody as described above. Calponin staining was carried out using the method and the same calponin antibody as described by Rizek and Kawaja [11]. Cells were prefixed in 4% paraformaldehyde for 10 min followed by incubation with the cell surface marker p75^NTR ^and its class specific fluorochrome-conjugated antibody as described above. Cells were then fixed in 100% ice cold methanol for 10 min followed by pretreatment with 20 μg/ml of proteinase K for 7 min and lastly incubated with calponin for 1 hr and its class specific antibody for 30 min. Hank's solution (Gibco) containing 5% calf serum and Hepes buffer was used to dilute the antibodies and for washing between incubations. In some experiments preincubation with proteinase K was omitted. Primary antibodies include monoclonal mouse anti-human Calponin (1:50; IgG1, Dako, Ely, UK). To identify OECs we used monoclonal mouse anti- and polyclonal rabbit anti-p75^NTR ^(hybridoma, 1:1; IgG1, [51], anti-rabbit, 1:800; Abcam, Cambridge, UK) respectively; and rabbit polyclonal anti-S100β [52] (1: 200; Dako). For identifying ORNs we used monoclonal mouse anti-βIII tubulin (TUJ1, 1:100; IgG1, Sigma). Connective tissue was identified by polyclonal rabbit anti-human fibronectin (1:100, Dako); mouse monoclonal anti-alpha smooth muscle actin (α-SMA, 1:400; IgG1, Chemicon, Hampshire, UK; [35] and mouse monoclonal Thy1.1/CD90 (1:100; IgG1, Serotec). Visualization of the primary antibodies was carried out using the class specific antibodies conjugated with fluorescein (green, FITC) or rhodamine (red, TRITC) (Southern Biotechnique, 1:100). Coverslips were mounted with VectorShield (Vector laboratories, Peterborough, UK) containing 4'6-diamidine-2-phenylindole-dihydrochloride (DAPI) to visualize the nuclei. Images were examined either by confocal microscopy (Olympus FV-1000) or by fluorescence microscopy (Zeiss Axioskop) using MetaMorph image analysis from Molecular Devices.

### Tissue preparation and immunohistochemistry

The olfactory mucosa and olfactory bulbs were dissected from the heads of postnatal day 7 Sprague-Dawley rat pups. The tissue from the P7 rats and the heads from embryonic E17-E20 pups were immersed in 4% paraformaldehyde for 1–3 days and then cryopreserved in 30% sucrose solution until equilibration. Sections of 10–20 μm were cut using a Bright cryostat onto Vectabond (Vector Lab) coated slides. For immunohistochemistry, the slides were washed 3 times for 5 min in PBS then treated with proteinase K 20 μg/ml for 7 min, washed and incubated in a blocking solution containing 10% goat serum and 0.3% triton-X 100 (Sigma) in PBS for 1 hr at RT. Sections were then incubated in the primary antibodies described above diluted in PBS-4%BSA for 2 hr at RT. After 3 washes of 5 min with PBS the sections were incubated with the appropriate FITC/TRITC conjugated anti-mouse or anti-rabbit secondary 1:100 (Southern Biotechnique, Cambridge Bioscience, Cambridge, UK) for 1 hr. After the incubation the sections were washed 3 times for 5 min with PBS and then mounted with VectorShield containing DAPI.

### Confrontation assays

Confrontation assays between OECs and astrocytes and Schwann cells and astrocytes were set up as previously described [17,18]. Briefly, 10-μl strips containing 10,000 cells of either OECs or Schwann cells were set up opposing a parallel 10-μl strip containing 10,000 astrocytes on a PLL-coated coverslip. Cells were allowed to attach for 1 hr before washing in DMEM-FBS to remove non-attached cells. Cultures were then maintained in DMEM-FBS, and allowed to grow towards each other over a period of 10 days, giving time for cells to make contact and interact. Cultures were then immunolabeled using antibodies to GFAP and p75^NTR ^or GFAP and calponin and mounted with VectorShield containing DAPI to visualize nuclei. Images were examined using fluorescence microscopy (Zeiss Axioskop) using MetaMorph image analysis from Molecular Devices. Because we can induce i) OECs to form boundaries with astrocytes or ii) Schwann cells to mingle with astrocytes by modifying the FGF/heparin sulphate proteoglycan signaling cascade; it is unlikely that the different, initial, long-term culture conditions for OECs and Schwann cells mediate the differential behavior in the confrontation assays [21]. Furthermore, similar differences in the way OECs and Schwann cells form boundaries with astrocytes have been demonstrated in vivo [21].

## Authors' contributions

MT carried out the immunohistochemistry of olfactory mucosal tissue, AS-S generated cultures for immunostaining. SCB carried out the immunocytochemistry. MT and SCB conceived the study and participated in its design and coordination and MT, ES, AS-S and SCB helped draft the manuscript. All authors read and approved the manuscript.
